# Deep learning reconstruction improves detection of focal liver lesions in hepatobiliary phase compared to conventional EOB-MRI

**DOI:** 10.3389/fmed.2026.1797117

**Published:** 2026-05-01

**Authors:** Xuewen Peng, Qichao Cheng, Runzhe Tian, Linlin Lang, Cheng Li, Ruixin Tao, Tianyong Xu, Dmytro Pylypenko, Liping Zuo, Dexin Yu, Weiwei Lv

**Affiliations:** 1 Department of Radiology, Qilu Hospital of Shandong University, Jinan, Shandong, China; 2 Division of Bariatric and Metabolic Surgery, Department of General Surgery, Qilu Hospital of Shandong University, Jinan, China; 3 GE Healthcare, Beijing, Daxing District, Beijing, China; 4 Qilu Medical Imaging Research Institute, Shandong University, Jinan, China

**Keywords:** deep learning reconstruction, focal liver lesions, Gd-EOB-DTPA-enhanced liver MRI, HCC, MRI

## Abstract

**Objective:**

In recent years, the application of Deep Learning Reconstruction (DL Recon) technology in MRI has significantly improved the detection rate of small focal liver lesions (FLLs). This study aims to compare the detection performance of DL Recon EOB-MRI with traditional Gd-EOB-DTPA-enhanced liver MRI during the hepatobiliary phase (HBP) for FLL detection.

**Methods:**

This prospective, single-center study included 53 patients who underwent EOB-MRI during the hepatobiliary phase (HBP). For each patient, four types of images were acquired: 3 mm thickness non-DL reconstruction (Standard Non-DL), 3 mm thickness DL reconstruction (Standard DL), 1 mm thickness non-DL reconstruction (HR Non-DL), and 1 mm thickness DL reconstruction (HR-DL). Lesions were categorized based on signal intensity into high-signal and low-signal types, and classified into five size groups: >30 mm, 20–30 mm, 10–20mm, 5–10mm, and <5 mm. Three abdominal radiologists independently assessed the number of FLLs on HBP images. Pairwise comparisons between the four types of images were made using the Wilcoxon signed-rank test and Generalized Estimating Equations to assess differences.

**Results:**

The intraclass correlation coefficient (ICC) between all groups was >0.90, indicating excellent consistency (*P* < 0.001). For high-signal lesions, no differences were observed across groups (except for the total number of high-signal lesions: Standard DL vs. Standard Non-DL: P†† = 0.048, all other *P* > 0.05). For large low-signal lesions (>10 mm), no significant differences were found between groups (*P* > 0.05). For small low-signal lesions (5–10 mm), the Standard DL group showed significantly more lesions than the Standard Non-DL group (*P*†† = 0.030). For small low-signal lesions (<5 mm), both DL and Non-DL HR techniques detected significantly more lesions than Non-DL alone (*P*† ≤ 0.032).

**Conclusion:**

DL-Recon combined with MR techniques demonstrates significant clinical value in enhancing the detection of small low-signal lesions in EOB-MRI hepatobiliary phase, significantly improving the detection rate of FLLs.

## 1 Introduction

In recent years, with the advancement of imaging technology, the detection rate of focal liver lesions (FLLs) has significantly improved. FLLs range from benign lesions (such as hemangiomas, focal nodular hyperplasia) to malignant lesions (such as hepatocellular carcinoma, metastatic tumors) (1). Although most FLLs are benign and require no intervention, certain lesions (such as atypical nodules, early hepatocellular carcinoma) have clear potential for malignancy. Small lesions with a diameter of <10 mm often face challenges in detection and characterization due to the limitations of traditional imaging technology (2, 3).

Magnetic resonance imaging (MRI), with its superior soft tissue resolution (down to sub-millimeter level), especially with Gd-EOB-DTPA-enhanced imaging during the hepatobiliary phase, can significantly improve the detection and characterization of small FLLs (with sensitivity exceeding 90%) (3). However, traditional MRI still faces bottlenecks: to obtain high signal-to-noise ratio (SNR) images, scan time needs to be extended (typically 15–20 min for the hepatobiliary phase), which can result in respiratory motion artifacts (occurring in 20%−30% of cases) (4), ultimately affecting lesion visibility.

In recent years, the introduction of Deep Learning Reconstruction (DL Recon) technology has provided a new direction for the innovation of MRI technology. This technique optimizes the image reconstruction algorithm to shorten scan times while improving image SNR and significantly reducing motion artifacts, thereby improving lesion detection rate and exam success rate (5).

However, most existing literature focuses on the application of DL Recon in conventional MRI sequences (6, 7). There is still a significant gap in research comparing its performance in EOB-MRI hepatobiliary phase imaging for detecting different sizes of FLLs, particularly when compared with traditional reconstruction methods. Therefore, this study systematically compares the detection performance of DL Recon EOB-MRI and traditional EOB-MRI in the hepatobiliary phase for FLL detection, focusing on the interaction between lesion size (diameter: ≤ 5 mm, 5–10 mm, 10–20 mm, 20–30 mm, ≥30 mm) and reconstruction technique. The results will provide a theoretical basis for the optimization of DL Recon parameters and lay a foundation for its precise application in early screening of liver cancer and monitoring high-risk populations.

## 2 Materials and methods

### 2.1 Study population

This study is a prospective study approved by the Ethics Committee of Shandong University (Ethics No. KYLL-202408- 031) with informed consent obtained from all participants. All methods were performed in accordance with the relevant guidelines and regulations, including the Declaration of Helsinki. This study included 53 patients (≥18 years) with focal liver lesions scheduled to undergo Gd-EOB-DTPA enhanced MRI using a 3-T scanner (SIGNA Architect, GE Healthcare) between January and December 2024. Patients were excluded if they were pregnant, had severe liver dysfunction, or had a history of allergic reactions to contrast agents.

### 2.2 MRI acquisition

For EOB-MRI image analysis, T2-weighted imaging (T2WI) during breath-holding and T1-weighted imaging (T1WI) during inspiration and expiration are required. Simultaneously, dynamic contrast-enhanced sequences are acquired, including the arterial phase (20–25 s), portal venous phase (approximately 1 min), and transition phase (3–5 min), combined with diffusion-weighted imaging (DWI) and hepatobiliary phase images (approximately 20 min). The entire examination process takes approximately 25 min. For each patient, four types of images were acquired: 3-mm thickness non-DL reconstruction (Standard Non-DL), 3-mm thickness DL reconstruction (Standard DL), 1-mm thickness non-DL reconstruction (HR Non-DL), and 1-mm thickness DL reconstruction (HR-DL). Deep learning reconstruction was performed using the vendor-provided technique (AIR™ Recon DL^®^, GE Healthcare). Lesions were categorized based on signal intensity into high-signal and low-signal types and further divided into five size groups: >30 mm, 20–30 mm, 10–20 mm, 5–10 mm, and <5 mm (The scanning parameters are presented in Table 1).

**Table 1:** Gd-EOB-DTPA enhanced MRI scanning parameters.

Thickness (mm)	TR (ms)	TE (ms)	FOV (mm^2^)	Scanning matrix	Slice number
3	3.8	1.6	400 × 360	384 × 260	140~160
1	3.8	1.6	400 × 360	384 × 260	140~160

### 2.3 Analysis of DL Recon EOB-MRI

Deep learning reconstruction was performed using AIR™ Recon DL^®^ (version 29.1R04) with a medium reconstruction strength. The application of deep learning in Gd-EOB-DTPA-enhanced MRI image reconstruction involves several key steps aimed at improving image quality and enhancing lesion detectability. First, through training on large datasets, the model can extract important features from the images, such as edges, textures, and the location and shape of lesions. Based on this, the model performs lesion segmentation, accurately identifying lesion areas. Additionally, deep learning optimizes the image's SNR, including noise suppression, artifact removal, and resolution enhancement, effectively improving image quality. Ultimately, the optimized images display higher quality and clearer lesion boundaries, aiding clinicians in making more precise diagnoses.

### 2.4 Image analysis

All de-identified MR images were independently reviewed by three abdominal radiologists with several years of experience in abdominal MRI. The radiologists were blinded to the MRI acquisition techniques. The final lesion detection count was taken as the average result of the three radiologists. In cases of significant inter-reader discrepancies, a joint review was conducted, with disagreements resolved through consensus or adjudication. Radiologists perform analyses by delineating regions of interest (ROIs), comparing lesion signal intensities during the hepatobiliary phase, and integrating characteristic imaging features—such as mosaic patterns or central scars—to systematically diagnose and identify focal liver lesions (8).

Hepatocellular carcinoma (HCC) typically presents with non-rim arterial phase hyperenhancement, followed by washout on the portal venous phase, and hypointensity on the hepatobiliary phase (HBP). Metastatic liver lesions commonly demonstrate rim arterial phase hyperenhancement, peripheral washout, and marked hypointensity on HBP. For benign lesions, dysplastic nodules (DN) usually show no obvious arterial phase hyperenhancement and appear iso- or slightly hyperintense on HBP; regenerative nodules (RN) typically present as iso-intense on the arterial phase and iso- or hyperintense on HBP; focal nodular hyperplasia (FNH) demonstrates homogeneous arterial phase hyperenhancement, remains hyperintense on HBP, and shows no washout.

### 2.5 Follow-up protocol

**Figure 1:**
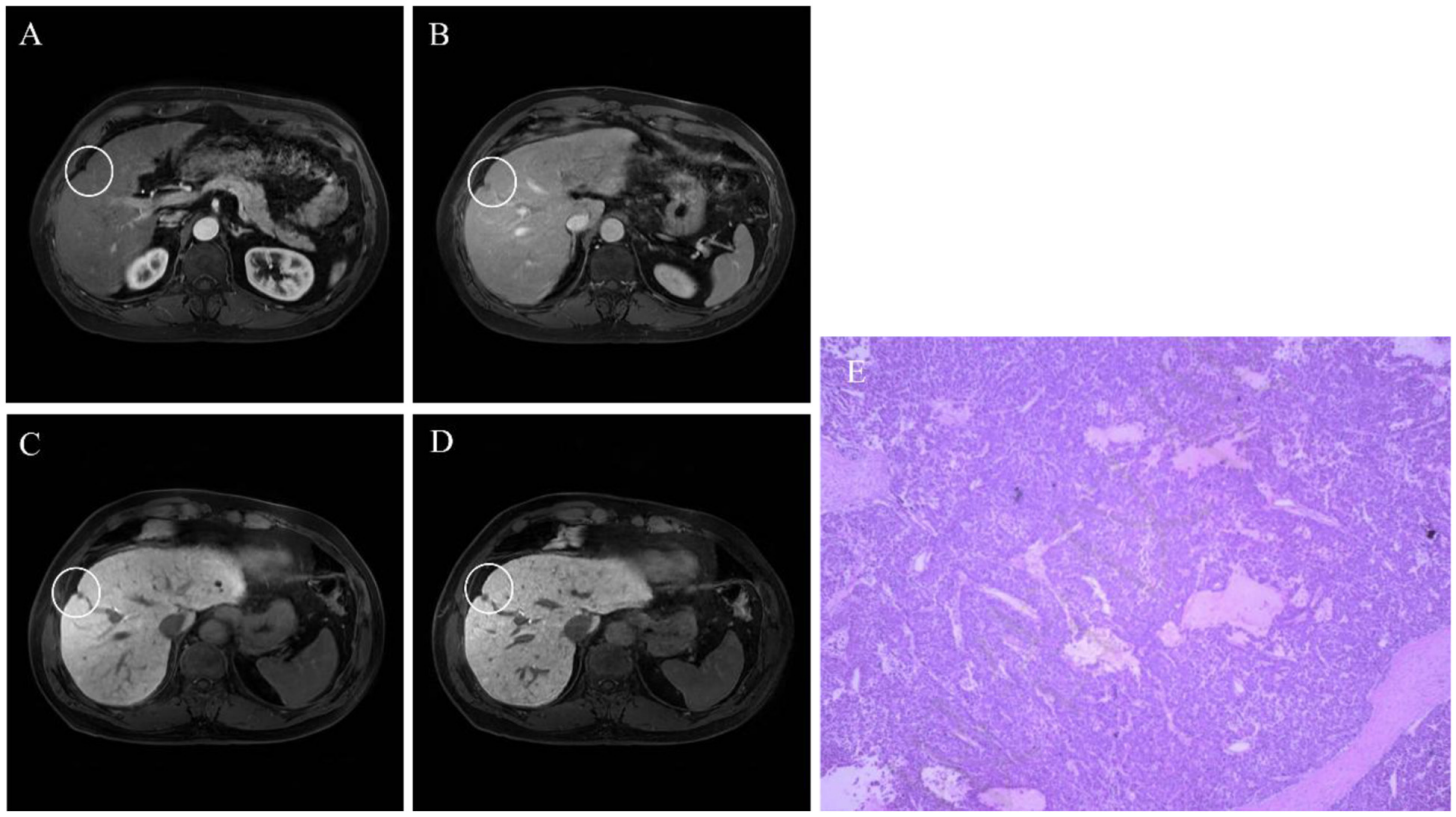
EOB-MRI demonstrated a newly detected 5 mm nodule. The lesion showed no obvious enhancement in the arterial phase **(A)** and exhibited rapid washout in the portal venous phase **(B)**. Small hypointense foci were observed in 3 mm **(C)** and 1 mm **(D)** HBP. Intraoperative pathology **(E)** confirmed trabecular-type HCC.

Lesions were classified as benign or malignant according to the Liver Imaging Reporting and Data System (LI-RADS) (9). In newly detected FLLs identified by EOB-MRI, lesions demonstrating overt malignant imaging features warrant modification of the existing treatment strategy. For resectable HCC, surgical resection within an appropriate extent is performed with informed consent, and the diagnosis is confirmed by intraoperative or postoperative histopathological examination (Figure 1). For unresectable HCC or liver metastases, necessary pathological evaluation is conducted to establish the diagnosis, followed by a comprehensive assessment incorporating the number, size, and distribution of newly detected lesions, the patient's general condition, and the control status of the primary lesions. Based on this assessment, subsequent treatment strategies are determined, including optimization or modification of systemic therapies (such as chemotherapy, targeted therapy, or immunotherapy), initiation or adjustment of locoregional treatments, and expansion of the radiotherapy field when indicated, with the aim of controlling tumor progression and delaying disease deterioration.

For benign lesions such as DN, RN, or FNH detected on EOB-MRI, close imaging follow-up (3–6 months) is recommended to monitor the evolution of the lesions. Particular attention should be paid to DN during follow-up; if imaging findings such as lesion enlargement or architectural distortion suggest an increased risk of malignant transformation, lesion resection with biopsy should be considered for further evaluation. Similarly, for hemangiomas, surgical resection with pathological confirmation is indicated if there is compression of adjacent structures or intralesional hemorrhage. For small lesions (<5 mm) with indeterminate nature, regular follow-up examinations are also recommended.

### 2.6 Statistical methods

Continuous variables were described using means ± standard deviation (*x* ± s) and normality was tested using the Shapiro-Wilk test. Pairwise comparisons between the four types of images were made using the Wilcoxon signed-rank test to assess differences in lesion counts across different images. The Wilcoxon signed-rank test is suitable for non-parametric paired sample tests and can effectively handle data with non-normal distributions. To further control for potential confounding factors, pairwise comparisons were analyzed using Generalized Estimating Equations (GEE) to evaluate statistical differences in lesion counts across images. Lesion counts were compared between the four image types based on lesion signal intensity (high-signal, low-signal) and size (categories: >30 mm, 20–30 mm, 10–20 mm, 5–10 mm, <5 mm). Wilcoxon signed-rank tests were used to compare the differences in lesion counts between images, assessing consistency in lesion detection. In this study, GEE was used as the primary analytical method, as it accounts for within-subject correlations and is more appropriate for repeated measures data. The Wilcoxon test was performed as a supplementary non-parametric method to provide additional validation, given its robustness in the presence of skewed distributions or outliers. The intraclass correlation coefficient (ICC) was used to evaluate intra-group consistency for lesion signal intensity and size classification. ICC was used to evaluate inter-reader agreement among the three radiologists. ICC values >0.90 were considered excellent, 0.75–0.90 as good, 0.5–0.75 as moderate, and <0.5 as poor. Statistical analysis was performed using SPSS software (version 26.0, IBM Corp.). Two-tailed *p*-values ≤ 0.05 were considered statistically significant.

## 3 Results

### 3.1 Baseline data

This study initially enrolled 64 patients with focal hepatic lesions. After excluding seven cases due to incomplete Gd-EOB-DTPA-enhanced MRI protocols and four cases with Child-Pugh C liver dysfunction, the final cohort comprised 53 patients. Baseline analysis revealed: 40 males (75.5%) and 13 females (24.5%) with mean age of 51.7 years; 37 hepatitis B virus (HBV) carriers (69.8%) and 37 cirrhotic patients (69.8%) (Table 2). The three radiologists demonstrated good inter-reader agreement (ICC = 0.87).

**Table 2:** Clinical characteristics.

Clinical information (*n* = 53)		Statistical value
Age		51.7 ± 9.9(24~78)
Sex	Male	40(75.5%)
	Female	13(24.5%)
Cirrhosis	–	37(69.8%)
Alcoholism	–	25(47.2%)
Hepatitis virus infection	Hepatitis B infection	37(69.8%)
	Hepatitis C infection	0(0.0%)
	Other infections	1(1.9%)
Child-Pugh	A	48(90.6%)
	B	5(9.4%)

### 3.2 Comparison of high-signal and low-signal lesions between groups

**Figure 2:**
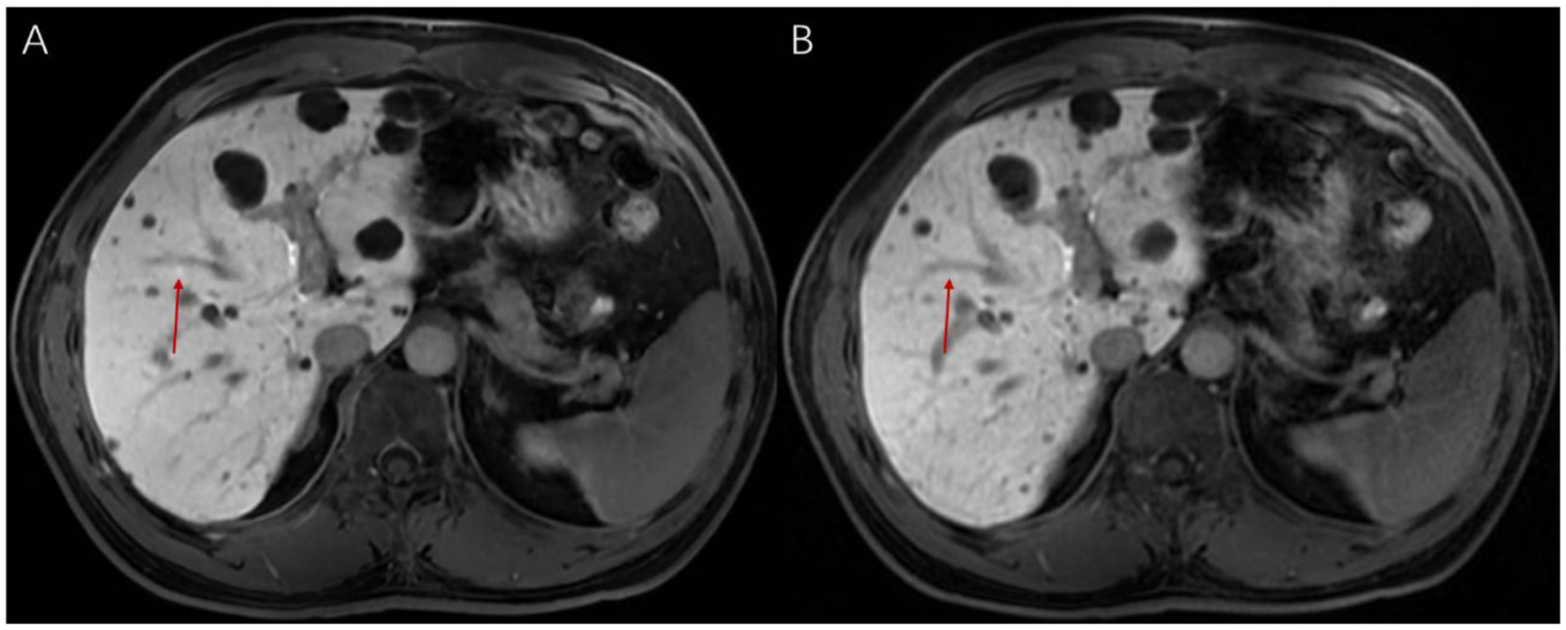
**(A)** Reconstruction results of high-signal lesions in EOB-MRI (slice thickness 3 mm) using deep learning techniques; **(B)** Imaging results of high-signal lesions in conventional EOB-MRI (slice thickness 3 mm). A regenerative nodule (RN), approximately 3.5 mm, is shown.

The analysis of high-signal lesions revealed some interesting trends. Due to differences in the statistical assumptions of the two methods, discrepancies were observed in some results presented in the table. Specifically, for the comparison between Standard DL and Standard Non-DL, inconsistency in statistical significance was found in the total number of high-signal lesions (*P*† = 0.076, *P*†† = 0.048), as well as in the number of low-signal lesions measuring 5–10 mm (0.056 vs. 0.030). For the comparison between Standard DL and Standard Non-DL, the total number of lesions was slightly higher in the Standard DL group (7.56 ± 18.31) compared to Standard Non-DL (7.22 ± 18.06). However, this difference was no statistically significant (*P*† = 0.076, *P*†† = 0.048). For lesions in the 5–10mm range, Standard DL showed a slight increase in the number of lesions (2.73 ± 7.99) compared to Standard Non-DL (2.60 ± 7.93), although this difference was not significant (*P*† = 0.081, *P*†† = 0.084). For lesions smaller than 5 mm, no significant differences were found (*P*† = 0.251, *P*†† = 0.257) (Figure 2) (Table 3).

**Table 3:** Comparisons of lesions numbers in HBP images.

Standard DL *vs*. standard Non-DL						HR DL *vs*. HR non-DL				
Variables	Standard DL	Standard Non-DL	Difference-a	*P*^†^ value	*P*^††^ value	HR DL	HR Non-DL	Difference-b	*P*^†^ value	*P*^††^ value
The number of high signal lesions			
Total	7.56 ± 18.31	7.22 ± 18.06	0.32 ± 1.33	0.076	0.048	8.36 ± 19.46	8.19 ± 19.30	0.17 ± 0.69	0.109	0.084
>30 mm	0.05 ± 0.23	0.05 ± 0.23	0.00 ± 0.00	NA	1.000	0.06 ± 0.23	0.06 ± 0.23	0.00 ± 0.00	NA	1.000
20–30 mm	0.07 ± 0.33	0.07 ± 0.33	0.00 ± 0.00	NA	1.000	0.09 ± 0.45	0.08 ± 0.33	0.02 ± 0.12	0.313	0.317
10–20 mm	0.69 ± 2.95	0.51 ± 2.45	0.15 ± 0.79	0.115	0.066	0.55 ± 2.33	0.57 ± 2.54	0.05 ± 0.27	0.654	0.655
5–10 mm	2.73 ± 7.99	2.60 ± 7.93	0.14 ± 0.49	0.081	0.084	2.96 ± 8.24	2.89 ± 8.14	0.12 ± 0.57	0.390	0.461
<5 mm	4.04 ± 10.03	3.98 ± 10.05	0.08 ± 0.32	0.251	0.257	4.70 ± 10.79	4.58 ± 10.82	0.09 ± 0.42	0.075	0.059
The number of low signal lesions			
Total	10.85 ± 18.37	9.87 ± 17.36	0.91 ± 2.98	0.025	0.007	12.23 ± 19.64	11.96 ± 19.70	0.39 ± 0.943	0.081	0.083
>30 mm	0.40 ± 0.87	0.38 ± 0.85	0.02 ± 0.12	0.313	0.317	0.42 ± 0.89	0.42 ± 0.89	0.00 ± 0.00	NA	1.000
20–30 mm	0.51 ± 0.92	0.49 ± 0.84	0.05 ± 0.21	0.563	0.564	0.49 ± 0.91	0.49 ± 0.91	0.00 ± 0.00	NA	1.000
10–20 mm	0.91 ± 2.39	0.95 ± 2.42	0.03 ± 0.25	0.313	0.317	1.06 ± 2.63	1.11 ± 3.00	0.08 ± 0.40	0.362	0.414
5–10 mm	3.84 ± 7.94	3.11 ± 6.93	0.73 ± 2.58	0.056	0.030	4.21 ± 8.67	4.13 ± 8.65	0.15 ± 0.56	0.390	0.527
<5 mm	5.20 ± 9.85	4.95 ± 9.34	0.39 ± 1.90	0.369	0.776	6.06 ± 10.51	5.81 ± 10.52	0.26 ± 0.71	0.025	0.032

*P*† value was used Generalized estimating equations.

*P*†† value was uesed Wilcoxon signed-rank test.

**Figure 3:**
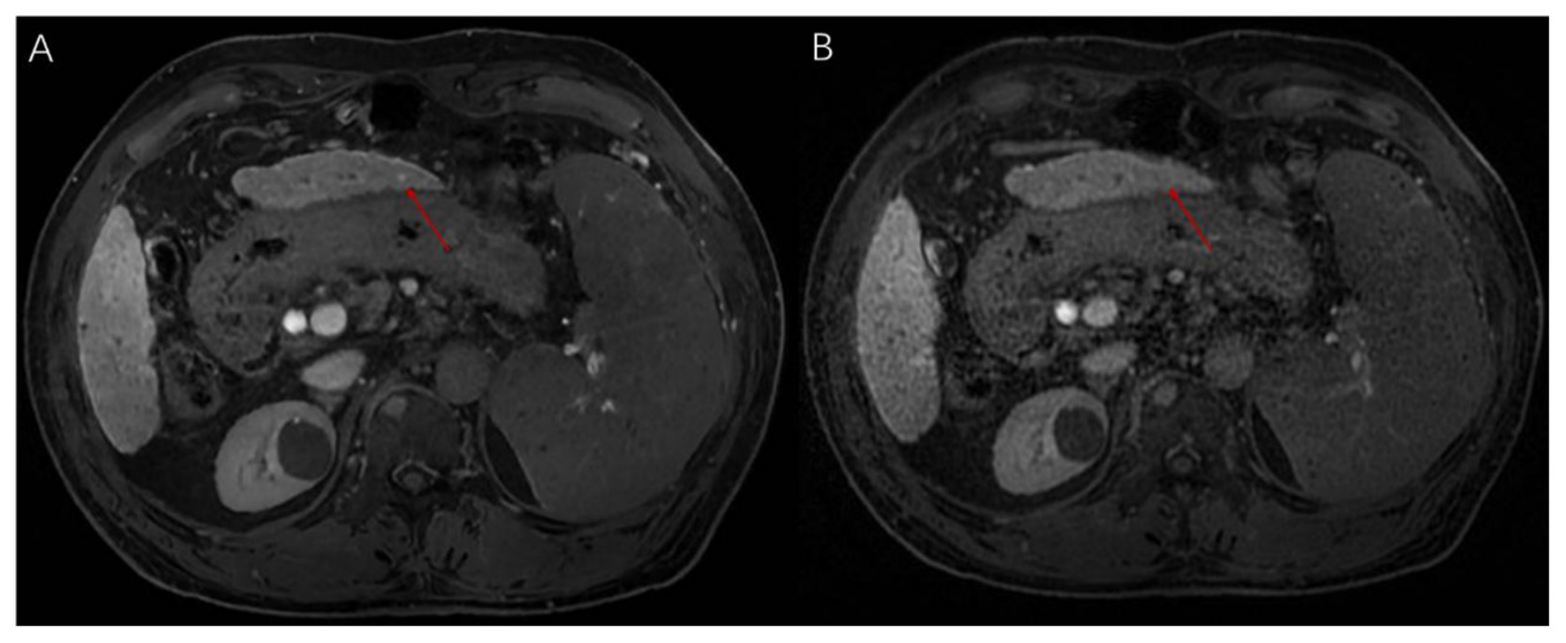
**(A)** Reconstruction results of high-signal lesions in EOB-MRI (slice thickness 1 mm) using deep learning techniques; **(B)** Imaging results of high-signal lesions in conventional EOB-MRI (slice thickness 1 mm). A regenerative nodule (RN), approximately 3.5 mm, is shown.

The comparison between HR DL and HR Non-DL showed that the total number of lesions was slightly higher in the HR DL group (8.36 ± 19.46) compared to HR Non-DL (8.19 ± 19.30), but again, the difference was not significant (*P*† = 0.109, *P*†† = 0.084). For lesions smaller than 5 mm, HR DL showed a higher mean (4.70 ± 10.79) compared to HR Non-DL (4.58 ± 10.82), although this difference was also not statistically significant (*P*† = 0.075, *P*†† = 0.059). Comparing Standard DL to HR DL, the total number of lesions was slightly higher in HR DL (8.36 ± 19.46) than in Standard DL (7.56 ± 18.31), but no significant difference was observed (*P*† = 0.296, *P*†† = 0.634). When analyzing lesions in the 20–30mm range, HR DL again showed slightly more lesions (0.09 ± 0.45) than Standard DL (0.07 ± 0.33), but the difference was not statistically significant (*P*† = 0.313, *P*†† = 0.317) (Figure 3).

**Figure 4:**
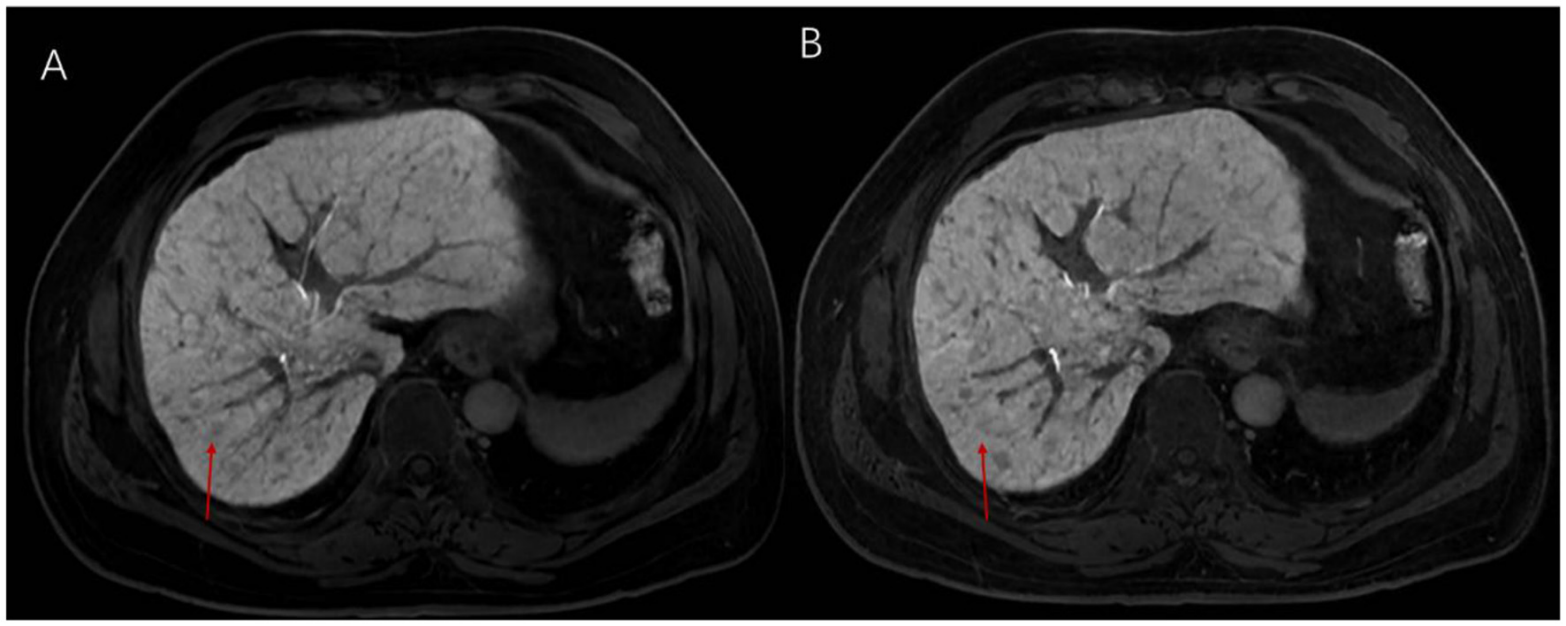
**(A)** Reconstruction results of EOB-MRI images (slice thickness 3 mm) for low signal lesions using deep learning techniques; **(B)** Conventional EOB-MRI imaging results (slice thickness 3 mm) for low signal lesions. A dysplastic nodule (DN), approximately 4 mm, is shown.

In contrast, the analysis of low-signal lesions showed more significant findings. For Standard DL vs. Standard Non-DL, the total number of lesions was significantly higher in the Standard DL group (10.85 ± 18.37) compared to Standard Non-DL (9.87 ± 17.36) (*P*† = 0.025, *P*†† = 0.007). When focusing on lesions in the 5–10 mm range, Standard DL showed a higher number of lesions (3.84 ± 7.94) compared to Standard Non-DL (3.11 ± 6.93). However, this difference was not statistically significant based on GEE analysis (*P*† = 0.056), while a significant difference was observed in the Wilcoxon test (*P*†† = 0.030) (Figure 4).

**Figure 5:**
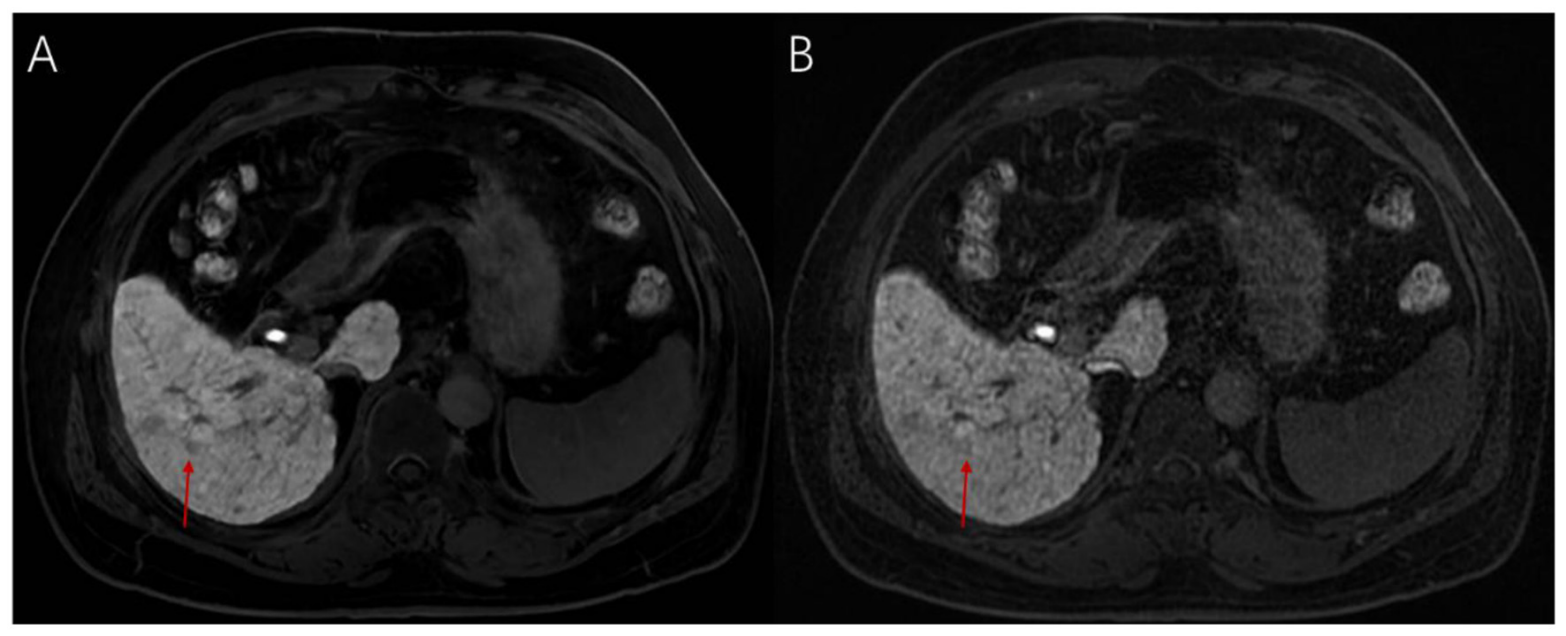
**(A)** Reconstruction results of EOB-MRI images (slice thickness 1 mm) for low signal lesions using deep learning techniques; **(B)** Conventional EOB-MRI imaging results (slice thickness 1 mm) for low signal lesions. A dysplastic nodule (DN), approximately 4 mm, is shown.

For the comparison between HR DL and HR Non-DL, HR DL showed significantly more lesions for those smaller than 5 mm (6.06 ± 10.51) compared to HR Non-DL (5.81 ± 10.52) (*P*† = 0.025, *P*†† = 0.032). Furthermore, the total number of lesions was significantly higher in HR Non-DL (11.96 ± 19.70) compared to Standard Non-DL (9.87 ± 17.36) (*P*† = 0.015, *P*†† = 0.021). This trend continued for lesions in the 5–10mm range, where HR Non-DL had significantly more lesions (4.13 ± 8.65) compared to Standard Non-DL (3.11 ± 6.93) (*P*†† = 0.031) (Figure 5).

### 3.3 Comparison of differences between groups

**Figure 6:**
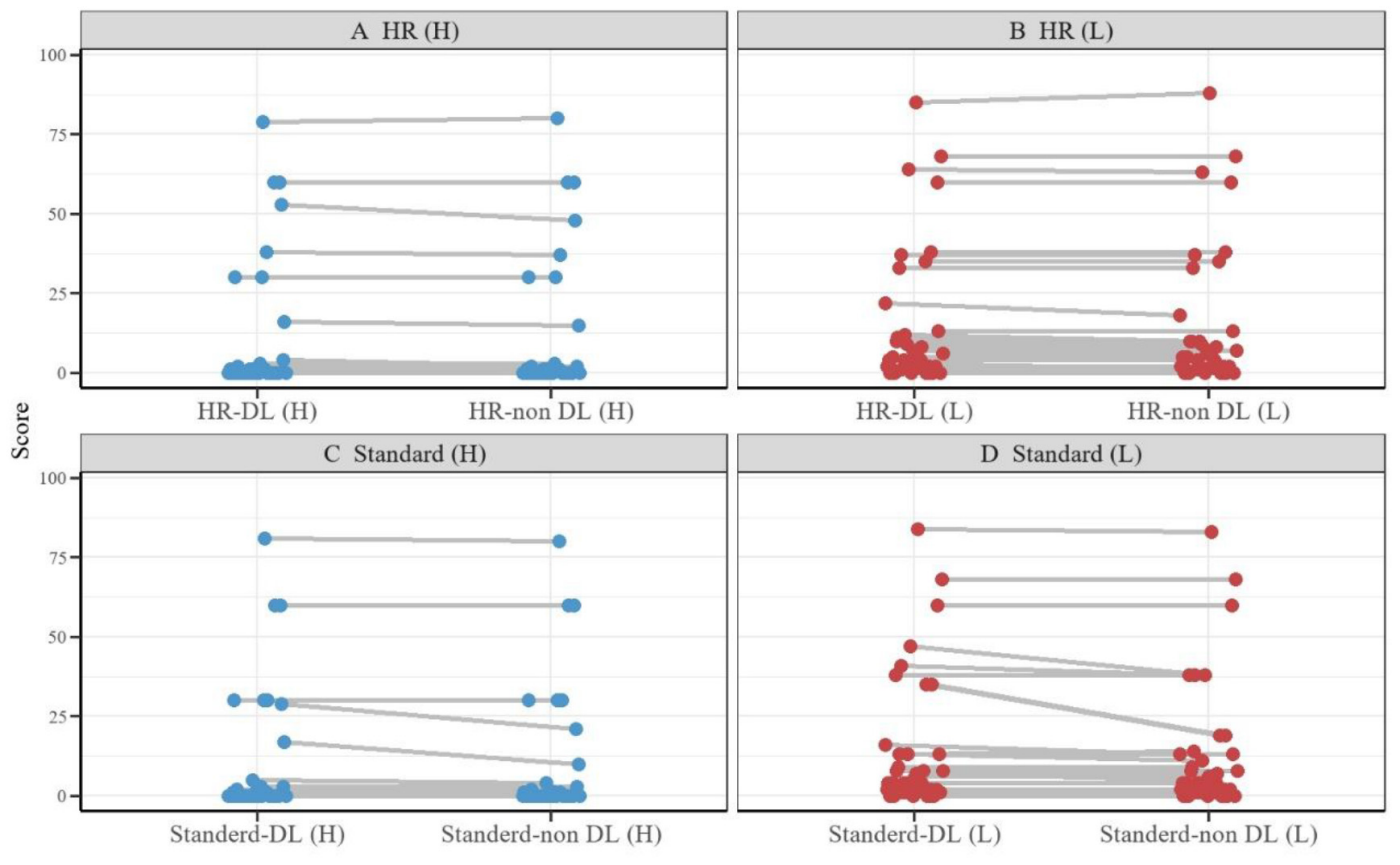
Paired dot plots. **(A)**. Difference of high signal lesions between HR DL and HR Non-DL, **(B)**. Difference of low signal lesions between HR DL and HR Non-DL, **(C)**. Difference of high signal lesions between Standard DL and Standard Non-DL, **(D)**. Difference of low signal lesions between Standard DL and Standard Non-DL.

Difference-a represents the absolute value of the difference between Standard DL and Standard Non-DL, Difference-b represents the absolute value of the difference between HR DL and HR Non-DL, and so on (Figure 6). In the analysis of lesion quantity, the intergroup differences exhibited the following characteristics: For low-signal lesions, the mean value of Difference Group A (0.32±1.33) showed an increasing trend compared to Difference Group B (0.17±0.69). A similar trend was observed in the comparison of high-signal lesions, where the value of Difference Group A (0.91±2.98) was higher than that of Difference Group B (0.39±0.94). However, these intergroup differences did not reach statistical significance (*P* = 0.748; *P* = 0.651) (Table 4).

**Table 4:** The difference between the absolute values of the difference in lesions numbers in HBP images.

Variables	Difference-a	Difference-b	*P-*value
The number of high signal lesions		
Total	0.32 ± 1.33	0.17 ± 0.69	0.748
>30 mm	0.00 ± 0.00	0.00 ± 0.00	1.000
20–30 mm	0.00 ± 0.00	0.02 ± 0.12	0.317
10–20 mm	0.15 ± 0.79	0.05 ± 0.27	0.402
5–10 mm	0.14 ± 0.49	0.12 ± 0.57	0.529
<5 mm	0.08 ± 0.32	0.09 ± 0.42	0.996
The number of low signal lesions		
Total	0.91 ± 2.98	0.39 ± 0.943	0.651
>30 mm	0.02 ± 0.12	0.00 ± 0.00	0.317
20–30 mm	0.05 ± 0.21	0.00 ± 0.00	0.081
10–20 mm	0.03 ± 0.25	0.08 ± 0.40	0.315
5–10 mm	0.73 ± 2.58	0.15 ± 0.56	0.172
<5 mm	0.39 ± 1.90	0.26 ± 0.71	0.633

*P*-value was uesed Wilcoxon rank-sum test.

Difference-a represents the absolute value of the difference between Standard DL and Standard Non-DL.

Difference-b represents the absolute value of the difference between HR DL and HR Non-DL.

### 3.4 Consistency analysis between groups

For high-signal lesions, all ICC values across groups were >0.90, indicating excellent consistency (*P* < 0.001); for lesions >30 mm, ICC = 1.000, indicating perfect consistency. For low-signal lesions, all ICC values across groups were >0.90, indicating excellent consistency (*P* < 0.001). The ICC between HR-DL and HR Non-DL for total lesion counts was 0.998, which was superior to the standard methods (ICC = 0.982). For small lesions (<5 mm), the ICC between HR-DL and HR Non-DL was 0.997, significantly higher than the standard methods (ICC = 0.976). Between-group comparisons showed that the ICC for Standard Non-DL and HR-DL was relatively lower for low-signal lesions (ICC = 0.951), suggesting that increasing resolution requires combining deep learning to improve consistency (Table 5).

**Table 5:** Consistency evaluation of lesion number assessment by different image types in HBP.

Variables	Standard DL *vs*. standard non-DL			HR DL *vs*. HR non-DL		
	ICC	95% CI	*P*-value	ICC	95% CI	*P*-value
The number of high signal lesions			
Total	0.997	0.994,0.998	<0.001	0.999	0.999,1.000	<0.001
>30 mm	1.000	–	–	1.000	–	–
20–30 mm	1.000	–	–	0.940	0.898,0.965	<0.001
10–20 mm	0.948	0.913,0.970	<0.001	0.992	0.986,0.995	<0.001
5–10 mm	0.998	0.996,0.999	<0.001	0.997	0.995,0.998	<0.001
<5 mm	0.999	0.999,1.000	<0.001	0.999	0.998,0.999	<0.001
The number of low signal lesions			
Total	0.982	0.968,0.990	<0.001	0.998	0.997,0.999	<0.001
>30 mm	0.988	0.979,0.993	<0.001	1.000	–	–
20–30mm	0.965	0.940,0.979	<0.001	1.000	–	–
10–20mm	0.994	0.989,0.996	<0.001	0.987	0.978,0.992	<0.001
5–10mm	0.924	0.872,0.955	<0.001	0.997	0.995,0.998	<0.001

## 4 Discussion

This study systematically compared the detection efficacy between conventional Gd-EOB-DTPA-enhanced MRI and deep learning-based EOB-MRI for focal hepatic lesions. Compared with conventional imaging methods, the combined application of Gd-EOB-DTPA-enhanced MRI and deep learning reconstruction technology can significantly improve the detection efficiency of focal hepatic lesions. Furthermore, the implementation of deep learning technology significantly improved diagnostic consistency during clinical evaluation.

This study demonstrates that deep learning reconstruction technology exhibits significant advantages in diagnosing hypointense lesions, enhancing detection sensitivity for various lesion types under different slice thickness conditions (1 mm/3 mm). The combined application of EOB-MRI and deep learning technology significantly increased the total number of detected lesions (10.85 ± 18.37; 11.96 ± 19.70), with a particularly notable improvement in the detection of small nodules (<5 mm) (3.84 ± 7.94; 4.13 ± 8.65). This technology combination effectively enhances the identification of small lesions, providing more sensitive imaging support for early intervention in tumor progression and other pathological changes. Interestingly, the advantages did not reach statistical significance in hyperintense lesions (*P* = 0.076, *P* = 0.109). This may be attributed to radiologists' inherently high sensitivity in visually identifying hyperintense lesions, whereas hypointense lesions, due to their lower contrast with surrounding tissues, present inherent limitations in manual interpretation. Deep learning utilizes a hierarchical feature fusion mechanism to gradually abstract low-level feature representations into high-level semantic features, thereby enabling intelligent diagnosis of lesions (10, 11). After completing a series of image processing steps such as noise suppression, artifact correction, and resolution optimization, the contrast between lesion tissues (especially low-signal regions) and surrounding normal tissues in EOB-MRI images is significantly enhanced (12, 13). The application of the combined model significantly improved the detection accuracy of low-contrast hypointense lesions (*P* = 0.025, *P* = 0.015), providing a safeguard against the risk of missed focal hepatic lesions.

In the liver imaging field, Gd-EOB-DTPA (Primovist), a hepatocyte-specific contrast agent, selectively enhances normal liver tissue and highlights lesion regions with low signal (14). It has become an essential tool for diagnosing focal liver lesions (FLL) (15). Studies show that Gd-EOB-DTPA-enhanced MRI has significantly higher sensitivity in detecting small FLLs (<1 cm in diameter) compared to conventional MRI, due to its unique hepatocyte uptake mechanism. After injection, the normal liver parenchyma enhances within 20 min, whereas lesions lacking hepatocyte function do not take up the contrast agent, creating a significant signal difference (16). However, conventional MRI techniques are limited by image noise, resolution, and motion artifacts, which may hinder precise evaluation of small lesions (17). This study innovatively combined DL reconstruction technology with Gd-EOB-DTPA-enhanced MRI, and the results demonstrated that AIR Recon DL-based EOB-MRI exhibited higher sensitivity in detecting small FLLs (especially lesions ≤ 10 mm and ≤ 5 mm). This breakthrough is attributed not only to the optimization of image quality by DL technology (e.g., Gibbs artifact suppression and improved SNR) (18) but also to the synergistic effect between Gd-EOB-DTPA and DL reconstruction (6, 19). DL technology strengthens the contrast boundary between lesions and liver parenchyma through high-resolution reconstruction (5), while Gd-EOB-DTPA further accentuates the signal differences between lesions and normal tissue through its specific uptake mechanism, providing multidimensional imaging support for the differential diagnosis of small FLLs (including morphological features, enhancement patterns, and dynamic signal evolution) (16). This combined strategy not only enhances diagnostic accuracy but also provides richer biological information (such as tumor vascularity and hepatocyte function) (20), aiding in distinguishing benign lesions (e.g., regenerative nodules) from malignant ones (e.g., early hepatocellular carcinoma or metastases) (21, 22).

Studies have shown that deep learning models exhibit excellent lesion recognition consistency in the diagnostic application of EOB-MRI, demonstrating high reliability in both hypointense lesions and hyperintense regions. Compared to traditional diagnostic methods that rely on subjective interpretation by multiple radiologists and are susceptible to interobserver variability (ICC = 0.976), especially with an increased risk of missed or misdiagnosed small lesions (diameter <5 mm), the deep learning model significantly enhances diagnostic consistency through objective quantitative analysis (ICC = 0.997) (23). The advantage of deep learning technology may stem from its ability to comprehensively capture lesion characteristics through extensive external data training, establishing refined diagnostic criteria that enable the effective recognition of subtle lesions that might be overlooked in conventional methods (11). The system enhances the distinguishability of lesions in reconstructed images by establishing a precise classification mechanism, significantly improving its ability to differentiate true lesions from artifacts and thereby reducing the risk of misdiagnosis (24). Combined with the contrast enhancement effect of Gd-EOB-DTPA-enhanced imaging in differentiating lesions from normal liver parenchyma (25), the synergistic application of deep learning and enhanced imaging technology establishes a stable and reliable diagnostic paradigm.

However, this study has some limitations. First, although the deep learning reconstruction algorithm (DL 1 mm) significantly improves the detection rate of focal liver lesions (FLLs) compared to conventional DL 3 mm scans, the potential risk of false positives in newly identified nodules has not been effectively validated. For lesions requiring surgical intervention, we recommend final diagnosis through histopathological examination (the gold standard). For cases requiring medical treatment or follow-up observation, a strict imaging follow-up protocol should be established, monitoring lesion volume changes or enhancement feature evolution to confirm biological characteristics. However, for lesions measuring <5 mm with indeterminate nature, as this study was conducted on a voluntary participation basis, variability in patient follow-up compliance made it difficult to perform systematic and long-term postoperative follow-up for all cases. For cases without pathological evidence and lacking comprehensive follow-up data, multimodal imaging fusion techniques (such as diffusion-weighted imaging and dynamic contrast-enhanced scans) and optimization of iterative reconstruction algorithms (such as denoising models based on GAN) should be employed to enhance diagnostic specificity.

## 5 Conclusion

The findings of this study demonstrate that DL-Recon combined with Gd-EOB-DTPA-enhanced MRI show significant clinical application value in detecting FLLs. Compared to conventional reconstruction methods, the DL-Recon technology substantially increases the detection rate of small lesion by optimizing image SNR and spatial resolution.

## Data Availability

The raw data supporting the conclusions of this article will be made available by the authors, without undue reservation.
